# Projected habitat contraction of *Camellia japonica* under climate change in China based on MaxEnt modeling

**DOI:** 10.3389/fpls.2026.1800676

**Published:** 2026-05-18

**Authors:** Rong Hu, Fang Li, Lu Wang, Fang Ji, Chaqin Tang, Chao Xu, Yanming Fang

**Affiliations:** 1 College of Tea and Food Science and Technology, Jiangsu Vocational College of Agriculture and Forestry, Zhenjiang, China; 2 Co-Innovation Centre for Sustainable Forestry in Southern China, and College of Life Science, Nanjing Forestry University, Nanjing, China; 3 School of Landscape Ecology, Ningbo City College of Vocational Technology, Ningbo, China

**Keywords:** *Camellia japonica* L, climate change, climatic factor, maxent modeling, potential distribution

## Abstract

**Introduction:**

The impact of climate change on the distribution of *Camellia japonica*, an economically important ornamental shrub, is a critical concern for conservation. This study aims to explore its potential geographic distribution under climate change in China.

**Methods:**

We used 56 carefully screened and spatially thinned occurrence records in a Maximum Entropy (MaxEnt) model. After filtering for multicollinearity, ten environmental variables were retained. Future projections were made for the SSP245 and SSP585 scenarios for the 2050s and 2070s.

**Results:**

The model showed high predictive accuracy (AUC = 0.958). Temperature factors, particularly Bio6 and Bio2, were the main determinants. Under current conditions, highly suitable habitats are mainly concentrated in eastern coastal regions. Future projections indicate a severe contraction of suitable habitats by the 2070s, with reductions of 80.1% and 90.9% under SSP245 and SSP585, respectively.

**Discussion:**

The findings suggest a severe habitat contraction for wild *C. japonica* populations under future climate scenarios, with limited evidence of a range shift. This highlights their vulnerability and the urgent need for targeted, spatially explicit conservation strategies.

## 1 Introduction

The geographical distribution of species is regulated by a variety of environmental factors, including soil composition, climate conditions, anthropological activities, and hydrological patterns (Soberón and Peterson, 2005). At a macro-geographical scale, climate factors play a dominant role in shaping the distribution patterns of organisms (Varol et al., 2021). The latest assessment by the Intergovernmental Panel on Climate Change (IPCC) shows that the global average temperature has risen by 1.09 C over the past 140 years (IPCC, 2021), and it is projected to increase by 0.3 C-4.5 C by 2100 (Stocker et al., 2013; Ma et al., 2022). Climate is the most significant global factor influencing the distribution of species and it can alter their distribution ranges (migration, expansion, contraction, or even extinction) (Li et al., 2013; Puchałka et al., 2021). A global temperature rise of 2 C could place 15%-35% of species at risk of extinction (Thomas et al., 2004), highlighting the urgent need to quantify the impacts of climate change on suitable habitats for key species.

Species Distribution Models (SDMs) are pivotal mathematical tools in biogeography and macroecology, designed to predict habitat suitability for species by correlating distribution data with environmental variables. These models evaluate species ecological requirements using environmental parameters extracted from occurrence records and the projection of potential distributions across spatiotemporal scales. As a cornerstone method in conservation biology and environmental sciences, SDMs support diverse applications, including invasive species management, endangered species protection, climate adaptation analysis, and land-use planning (Butler et al., 2016). Historically, SDMs evolved from early bioclimatic approaches (e.g., BioClim, Climex) to advanced algorithms, such as generalized linear/additive models and the Maximum Entropy (MaxEnt) model. Among these, the MaxEnt framework, introduced by Phillips et al. (2004), has emerged as a gold standard due to its robust tolerance for limited data, high predictive accuracy, and operational simplicity (Yan et al., 2020; Ma and Li, 2023). By maximizing entropy to infer species probability distributions, MaxEnt enables precise identification of ecological niches and adaptive ranges, making it indispensable for the forecasting of species responses to past, present, and future environmental shifts (Zhang et al., 2018). Its versatility has driven widespread adoption across multidisciplinary contexts, from pest control to conservation prioritization, solidifying its role as a premier analytical tool in spatial ecology.

*Camellia japonica* L., a Tertiary relict species of the family Theaceae, is native to East Asia (including southern China, Japan, and the Himalayas) and it is now widely distributed across tropical and subtropical regions worldwide (Pereira et al., 2022). Although *C. japonica* is distributed across East Asia (China, Japan, and Korea), the present study focuses on remnant wild populations within China, which represent the marginal and fragmented portion of its distribution and are of particular conservation concern. With its rich flower colors (red, white, purple, pink, and yellow), *C. japonica* holds both ornamental and economic value. In China, it has a cultivation history of over 2000 years, resulting in the generation of more than 800 cultivated varieties (Li et al., 2021; Guan et al., 2014). However, in stark contrast to its extensive cultivation, the wild populations of *C. japonica* have experienced severe declines due to long-term overharvesting and habitat destruction and they are now sparsely distributed only on coastal islands with minimal human disturbance (Fan et al., 2022). These remnant wild populations are invaluable germplasm resources, holding critical genetic information and adaptive potential. They play an irreplaceable role in supporting the species evolutionary resilience and ecosystem functions.

Although previous studies have focused on the physiological characteristics (Wang et al., 2020), chemical composition (Majumder et al., 2020; Pereira et al., 2023), and genetic mechanisms of *C. japonica*’s flower color (Zhang, 2008), systematic projections of the distribution dynamics for its remnant wild populations under future climate change scenarios are still lacking. Crucially, the potential for these populations to shift their geographic ranges in response to climate change remains unknown. Wild populations of *C. japonica*, with their narrow distribution and fragile habitats, are likely highly sensitive to climate changes. The loss of these wild populations would lead to irreversible genetic erosion. Therefore, climate suitability modeling for this species must be conducted to understand its ecological adaptive limits and to inform the delineation of priority conservation areas, the preservation of germplasm resources, and the development of adaptive management strategies.

Considering the very limited distribution of remnant wild populations of *C. japonica* in China, the accurate prediction of potential suitable habitats and discerning future migratory patterns are essential to address the impacts of climate change. To bridge this knowledge gap and provide a scientific basis for its conservation, this study aims to: (1) identify the crucial climatic variables that influence the distribution of suitable areas for *C. japonica*; (2) predict potential suitable regions for the colonization of *C. japonica* under extant and forthcoming global climate change scenarios, and (3) leverage predictions of future climate change to delineate the spatial dynamics of habitat suitability, thereby offering a scientific groundwork for the meticulous introduction, safeguarding, and propagation of *C. japonica*.

To achieve these objectives, we synthesized climate data and records of species occurrence to construct models of both the present and prospective distributions of *C. japonica* throughout diverse Chinese regions. The study employed the MaxEnt model to project three distinct climate scenarios across China, aligning with three different timeframes: current (1970-2020), the future 2050s (2041-2060), and 2070s (2061-2080).

## 2 Materials and methods

### 2.1 Study area

**Figure 1:**
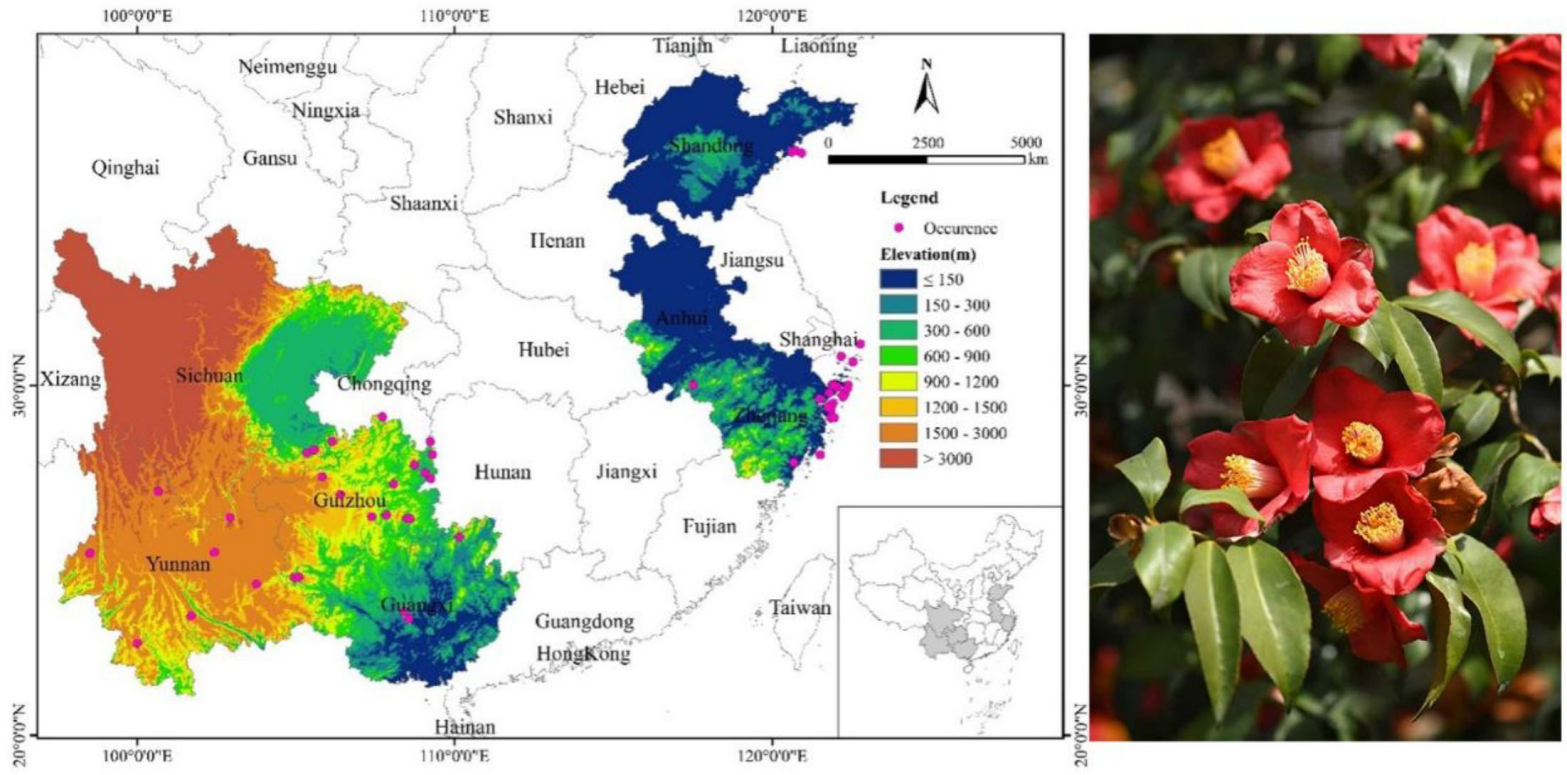
Spatial distribution of verified occurrence records of *Camellia japonica* used for MaxEnt modeling in China. Photo taken by co-author Lu Wang.

The study area was defined based on the spatial extent of occurrence records used for modeling (**Figure 1**). These records span multiple regions in China, including eastern coastal and southwestern areas. According to the Flora of China, the recognized native distribution of *C. japonica* in China is primarily restricted to Shandong, Zhejiang, and Taiwan. However, additional occurrence records have been reported in specimen databases and literature sources beyond this core range.

Therefore, the study area does not represent the strict floristic distribution of the species, but rather the environmental space defined by available occurrence data, which is commonly adopted in species distribution modeling to capture the realized climatic conditions associated with extant populations.

### 2.2 Collection of occurrence data

*C. japonica* specimens were excluded if there was incorrect identification, duplicate records, artificial introduction, and unclear information. From the remaining records, we applied a spatial thinning procedure with a minimum distance of 10 km (using the ‘spThin’ R package, Aiello-Lammens et al., 2015) to reduce spatial autocorrelation. A spatial thinning procedure (10 km) was applied to reduce sampling bias and spatial autocorrelation. After thinning, a total of 56 occurrence records at the county level were retained from specimen databases and the literature (**Figure 1**), mainly from the Chinese Virtual Herbarium (CVH, http://www.cvh.ac.cn/), National Specimen Information Infrastructure (NSII, http://www.nsii.org.cn/), the Global Biodiversity Information Facility (GBIF, http://www.gbif.org), Web of Science, and China National Knowledge Infrastructure (CNKI, http://oversea.cnki.net/tra).

To ensure that only wild populations were included, we implemented a conservative multi-step filtering strategy. First, each record was manually examined, and only those explicitly associated with natural habitats (e.g., “wild,” “natural forest,” “mountain slope,” or “native habitat”) were retained. Records indicating anthropogenic origin (e.g., “cultivated,” “planted,” or located in urban gardens and plantations) were excluded. Second, all retained records were cross-validated with authoritative floristic references, including regional floras and the Flora of China, to ensure consistency with the recognized native distribution range of the species. Records that could not be confidently assigned as natural occurrences were removed.

We acknowledge that distinguishing wild from cultivated individuals based solely on specimen metadata and literature descriptions may introduce some uncertainty, particularly in regions with a long history of cultivation. However, to minimize this risk, we adopted strict inclusion criteria and prioritized data reliability over sample size. This conservative approach reduces the likelihood of incorporating cultivated records that could bias ecological niche estimation. This regional focus inevitably limits the representation of the full ecological niche of the species; however, it allows for a more accurate characterization of the realized climatic niche of remnant wild populations within China.

Although we did not conduct a dedicated field survey for this study, all occurrence records were manually verified against specimen metadata and literature descriptions. A subset of records (approximately 30%) came from recent field-collected specimens with precise GPS coordinates, as indicated in the original data sources. This combination of herbarium and field-based records enhances the reliability of the occurrence dataset.

### 2.3 Environmental variables acquisition

We extracted 19 climatic variables from the WorldClim database (http://www.worldclim.org/) (Fick and Hijmans, 2017) as predictors for possible habitats of *C. japonica* at a spatial resolution of 2.5 arc minutes (**Table 1**) (Hijmans et al., 2005). We conducted research on three periods: the current climatological period (1970-2020), the future 2050s (2041-2060), and the future 2070s (2061-2080). The shared socioeconomic pathways (SSPs) of the Coupled Model Intercomparison Project Phase 6 (CMIP6) make future scenarios more reasonable than the representative concentration pathway (RCP) of the CMIP5 (Su et al., 2021; Sun et al., 2021). Thus, we selected future climatic data from five general circulation models (GCMs) available within the CMIP6 framework: BCC-CSM2-MR, CNRM-CM6-1, IPSL-CM6A-LR, MIROC6, and MRI-ESM2-0, predicted under SSP245 (medium emission scenario) and SSP585 (high emission scenario) future climate change scenarios. Two climate scenarios (SSP245 and SSP585) were selected to represent moderate and high greenhouse gas emission pathways, respectively. These scenarios are commonly used to capture a plausible range of future climate uncertainty. In addition, projections were conducted for the 2050s (2041–2060) and 2070s (2061–2080), which correspond to mid- and late-century timeframes frequently used in climate impact studies. These periods are particularly relevant for assessing long-term ecological responses and informing conservation planning. To reduce uncertainty associated with individual GCMs, we used the ensemble mean of these five models for all future projections (Su et al., 2021).

**Table 1:** Subset of environmental factors used in Maxent species distribution modeling.

Variable code	Bioclimatic variables description	Unit	Percent cumulative contribution	Permutation importance
**Bio1**	**Annual Mean Temperature**	**°C**	**1.78**	**21.25**
**Bio2**	**Mean Diurnal Range (mean of monthly (max temp-min temp))**	**°C**	**27.59**	**7.23**
Bio3	Isothermality (bio2/bio7) (×100)	%	–	–
Bio4	Temperature Seasonality (standard deviation ×100)	°C	–	–
**Bio5**	**Max Temperature of the Warmest Month**	**°C**	**5.92**	**10.29**
**Bio6**	**Min Temperature of the Coldest Month**	**°C**	**45.60**	**41.26**
Bio7	Temperature Annual Range (bio5-bio6)	°C	–	–
Bio8	Mean Temperature of the Wettest Quarter	°C	–	–
Bio9	Mean Temperature of the Driest Quarter	°C	–	–
**Bio10**	**Mean Temperature of the Warmest Quarter**	**°C**	**0.82**	**2.83**
Bio11	Mean Temperature of the Coldest Quarter	°C	–	–
**Bio12**	**Annual Precipitation**	**mm**	**0.39**	**0.87**
**Bio13**	**Precipitation of the Wettest Month**	**mm**	**0.35**	**1.29**
**Bio14**	**Precipitation of the Driest Month**	**mm**	**1.69**	**4.05**
**Bio15**	**Precipitation Seasonality (coefficient of variation)**	**%**	**8.22**	**1.42**
**Bio16**	**Precipitation of the Wettest Quarter**	**mm**	**7.63**	**9.50**
Bio17	Precipitation of the Driest Quarter	mm	–	–
Bio18	Precipitation of the Warmest Quarter	mm	–	–
Bio19	Precipitation of the Coldest Quarter	mm	–	–

The key environmental variables retained by the model are indicated in bold.

This study focuses on climatic variables because climate is the primary driver of species distributions at broad spatial scales. Although environmental factors such as soil, land cover, and topography may influence species occurrence at local scales, their inclusion in future projections is often constrained by the lack of consistent, high-resolution datasets across temporal scenarios. To ensure comparability between current and future predictions and to avoid potential model overfitting, we restricted the analysis to climatic variables. This approach is widely adopted in large-scale species distribution modeling studies. Topographic and edaphic factors were not included due to the lack of consistent, high−resolution future projections for these variables, and because their inclusion would reduce comparability across time periods and potentially introduce overfitting. This rationale follows previous large−scale SDM studies (e.g., Xie et al., 2021).

### 2.4 Environmental variable screening and selection

To enhance model reliability and interpretability by mitigating multicollinearity, a rigorous screening process was applied to the initial set of 19 bioclimatic variables (Gebrewahid et al., 2020). First, a Pearson correlation analysis was performed on all variables using R software (version 3.6.1). Variable pairs with a correlation coefficient |r| ≥ 0.8 were considered highly collinear (Puchałka et al., 2021). Within each highly correlated pair, the variable with the lower contribution to a preliminary MaxEnt model (run with default settings) was removed, prioritizing the retention of factors with greater explanatory power for the species’ distribution (Qin et al., 2017; Li et al., 2016).

After correlation-based filtering, a subset of 10 bioclimatic variables was retained for model construction (**Table 1**). To further assess potential multicollinearity, Variance Inflation Factor (VIF) values were calculated using the ‘usdm’ R package. VIF was used as a diagnostic indicator rather than a strict exclusion criterion. In addition, a subset of variables with VIF < 10 was identified and reported in **Supplementary Table S1** to demonstrate low multicollinearity conditions. This was provided for transparency rather than as a variable selection criterion. Although some of the retained variables exhibited relatively high VIF values, they were preserved due to their ecological relevance and contribution to model performance. Previous studies have shown that MaxEnt is relatively robust to multicollinearity among predictors (Phillips et al., 2006; Elith et al., 2011). Therefore, the final variable set represents a balance between statistical independence and ecological interpretability, and was used in all subsequent modeling analyses.

### 2.5 Model configuration and future climate data

The final species distribution models were built using MaxEnt software (version 3.4.3). MaxEnt was selected as the modeling algorithm due to its strong performance with presence-only data and its robustness when sample sizes are limited. Given that this study is based on 56 occurrence records, MaxEnt provides a reliable and interpretable framework for modeling species distributions. Although ensemble modeling approaches (e.g., Biomod2) can improve predictive robustness by integrating multiple algorithms, they typically require larger datasets and introduce additional model complexity. Therefore, a single-model approach was adopted to balance model interpretability and data constraints. To optimize model complexity and avoid overfitting, we performed model tuning using the ENMeval R package (Kass et al., 2021). We tested regularization multipliers of 0.5, 1, 1.5, 2, 2.5, 3, 3.5, 4, 4.5, 5, 5.5, 6, and feature class combinations (L, LQ, LQH, LQHP, LQHPT). The best model was selected based on the lowest AICc, which corresponded to a regularization multiplier of 0.5 and LQ features. This model was used for all final analyses. To account for potential spatial sampling bias and overfitting, a spatial block partitioning method (4-fold) was used for cross-validation.

For future projections, climate data for the 2050s (2041-2060) and 2070s (2061-2080) under SSP245 and SSP585 scenarios were obtained from five general circulation models (GCMs) within the CMIP6 framework: BCC-CSM2-MR, CNRM-CM6-1, IPSL-CM6A-LR, MIROC6, and MRI-ESM2-0. To reduce the uncertainty inherent in any single GCM, we utilized the ensemble mean of these five models to represent future climatic conditions (Su et al., 2021).

For future climate projections, we used the default clamping option in MaxEnt (version 3.4.3). Clamping constrains the values for each environmental variable in the projected climate layers to the minimum and maximum values observed in the training data for that variable. This approach prevents the model from extrapolating beyond the range of environmental conditions used for model calibration, thereby avoiding biologically unrealistic predictions when projecting to novel climates. To further reduce extrapolation uncertainty, we used the ensemble mean of five GCMs rather than relying on any single climate model.

### 2.6 Future potentially suitable distribution area

*C. japonica* occurrence data and 19 environmental variables were used to predict potentially suitable areas in the three periods by MaxEnt software (version 3.4.3, Phillips et al., 2021). We selected 75% occurrence data for training, and the remaining 25% was used for testing. The probability value of the future suitable area map generated by the model was between 0 and 1. The closer the value is to 1, the greater the probability of species existence. To define habitat suitability classes, we first determined a presence/absence threshold using the maximum Youden’s index (sensitivity + specificity -1), which maximizes the sum of sensitivity and specificity and is widely recommended for evaluating model-based binary classifications (Liu et al., 2016). The calculated threshold was approximately 0.18, which we rounded to 0.2 for practical application. This value was further supported by the minimum training presence threshold (0.19) and aligns with commonly used thresholds in species distribution modeling (Phillips and Dudík, 2008). Based on this binary threshold, we classified areas with predicted probability < 0.2 as unsuitable. For suitable areas (≥ 0.2), we further divided them into three categories-low (0.2-0.4), medium (0.4-0.6), and high (0.6-1.0)-following the equal-interval approach widely adopted in MaxEnt-based studies (Abolmaali et al., 2018; Gebrewahid et al., 2020). This classification balances ecological interpretability with consistency across climate scenarios.

The area under curve (AUC) is the area under the receiver operating characteristic curve (ROC) (Phillips and Dudík, 2008). In our study, it was used to determine the accuracy of the model, which was divided into five levels: excellent (0.9-1.0), good (0.8-0.9), average (0.7-0.8), poor (0.6-0.7), and failure (0.5-0.6) (Gebrewahid et al., 2020).

## 3 Results

### 3.1 Accuracy of the model

**Figure 2:**
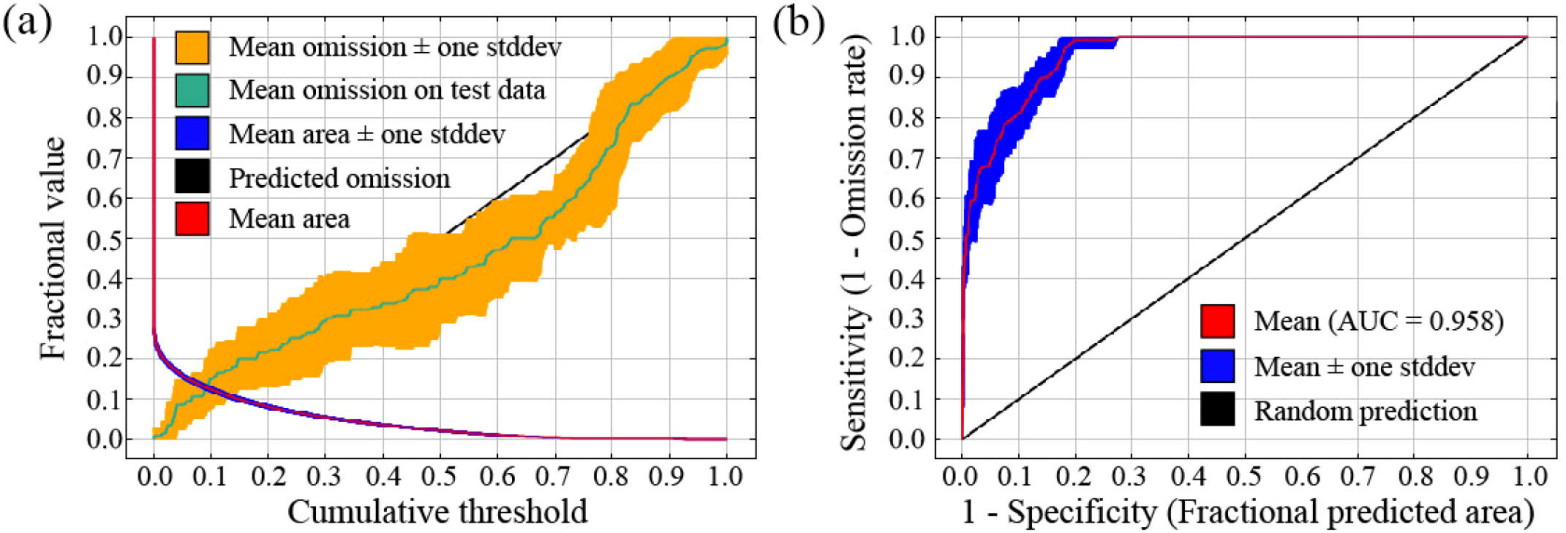
Omission rates versus predicted areas for *C. japonica*: **(a)** Area under the curve (AUC) for the receiver operating characteristic (ROC) analysis; **(b)** ROC curve and AUC value of the model.

The omission rate refers to the proportion of test localities that fall outside the pixels predicted as suitable for the species (Phillips et al., 2006), and a low omission rate is a necessary (but not sufficient) condition for a good model (Anderson et al., 2003). In **Figure 2a**, the red line indicates the mean area, and the black line the predicted omission rate (covered by the range of mean omission). The omission rates of the model training samples are shown by the light blue line (**Figure 2a**), which shows that the omission rate of the test set is consistent with the predicted omission rate. The results demonstrate that the model fitted the training data well, and the test data and training data were unique.

The ROC (receiver operating characteristic) curve for the reconstructed MaxEnt model is depicted in **Figure 2b**. Following 10 repetitions of training and testing, the mean AUC values were recorded at 0.960 for training and 0.958 for testing, respectively. Across the 10 replicates, the test AUC ranged from 0.938 to 0.979 with a standard deviation of 0.016, indicating stable and robust model performance. In addition to AUC, the True Skill Statistic (TSS) was calculated to further evaluate model performance. The TSS values were consistently high (TSS > 0.8), indicating strong agreement between observed and predicted distributions and confirming the robustness of the model. The maximum Youden’s index threshold for distinguishing suitable from unsuitable habitats was calculated as 0.18, confirming the ecological validity of using 0.2 as the cutoff for low suitability. An AUC metric exceeding 0.9 is indicative of an ‘excellent’ model (Phillips et al., 2006; Sun et al., 2020) and confirms the model’s exceptionally high precision in prediction. These results show that this model is well-equipped to identify suitable habitats for *C. japonica* in China.

### 3.2 Evaluating the contribution and influence of environmental variables

**Figure 3:**
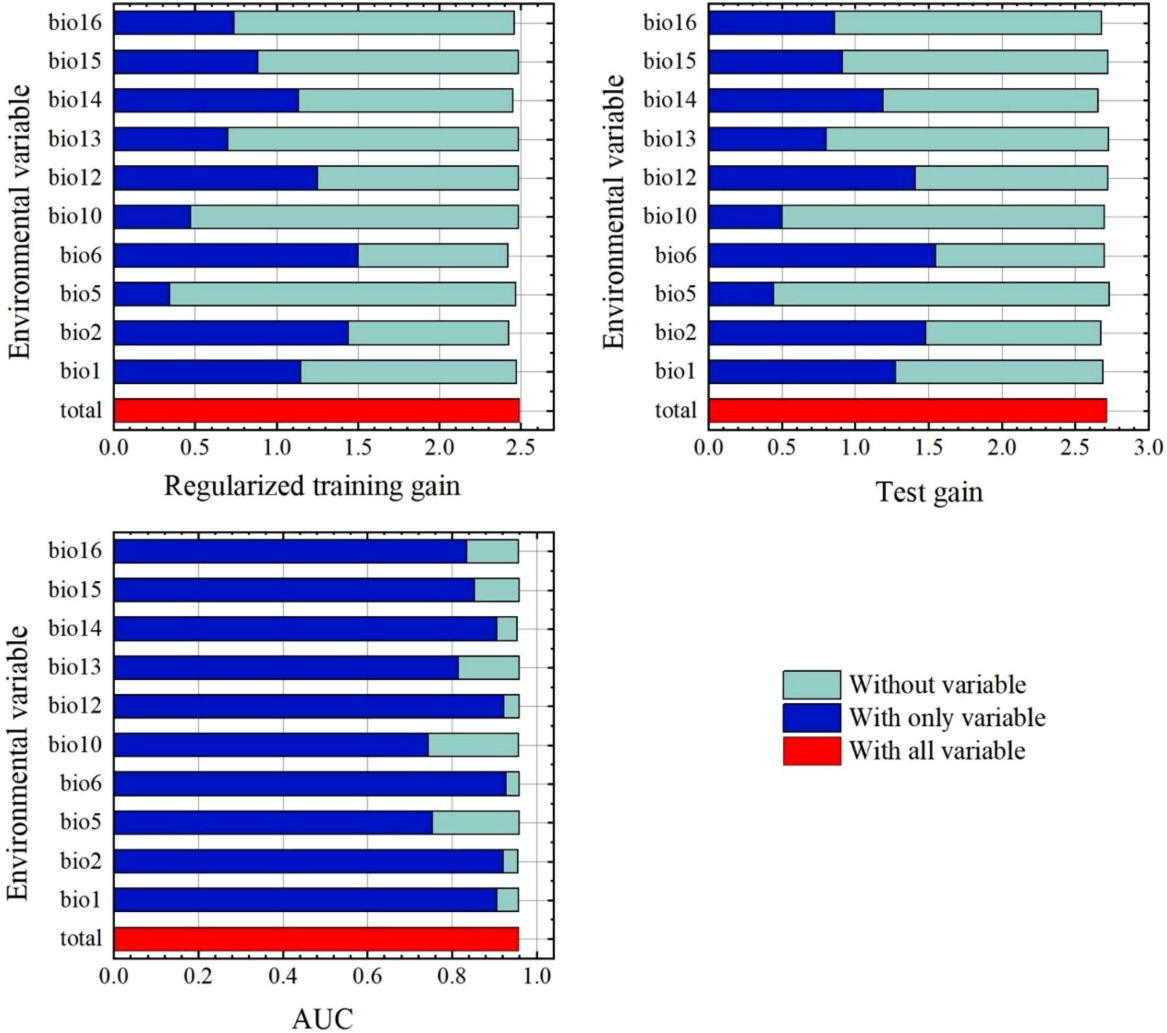
Jackknife test showing the importance of the selected environmental variables.

Among the 10 environmental variables used for model establishment, minimum temperature of the coldest month (Bio6), mean diurnal range (mean of monthly (max temp-min temp)) (Bio2), and annual precipitation (Bio12) all made good predictions, as shown in the variable importance jackknife test results and AUC (**Figure 3**). These three variables indicate a good fit with the data. However, maximum temperature of the warmest month (Bio5) and mean temperature of the warmest quarter (Bio10) performed worse than other environmental variables in regularized training gain, test gain, and AUC (**Figure 3**). Such statistical analysis indicated that these two variables were less important in predicting the current distribution of *C. japonica*. Regarding the relative contributions to the model (**Table 2**), the top three environmental variables in permutation importance were minimum temperature of the coldest month (Bio6), annual mean temperature (bio1), and maximum temperature of the warmest month (Bio5), with 41.26%, 21.25%, 10.29%, respectively. This indicated that temperature significantly affected species distribution (**Figure 2**; **Table 2**). Similar findings on temperature‑driven range constraints have been reported by Zhao et al. (2021). Meanwhile, the top three environmental variables in relative contributions were minimum temperature of the coldest month (Bio6), mean diurnal range (mean of monthly (max temp-min temp)) (Bio2), and precipitation seasonality (Bio15), with 45.60%, 27.59%, 8.22%, respectively.

**Table 2:** Area of suitable habitats for *C. japonica* under different future climate scenarios (km^2^).

Scenarios	Unsuitable area	Low suitable area	Middle suitable area	High suitable area	Total suitable area
Current	8,652,325	528,348	228,525	83,296	840,169 (8.8%)
SSP245 2050s	9,274,895	173,645	28,954	11,848	214,448 (2.2%)
SSP245 2070s	9,321,776	136,075	23,030	8,463	167,568 (1.7%)
SSP585 2050s	9,325,237	121,174	35,929	10,386	167,489 (1.7%)
SSP585 2070s	9,418,304	55,958	11,746	6,719	74,423 (0.8%)

**Figure 4:**
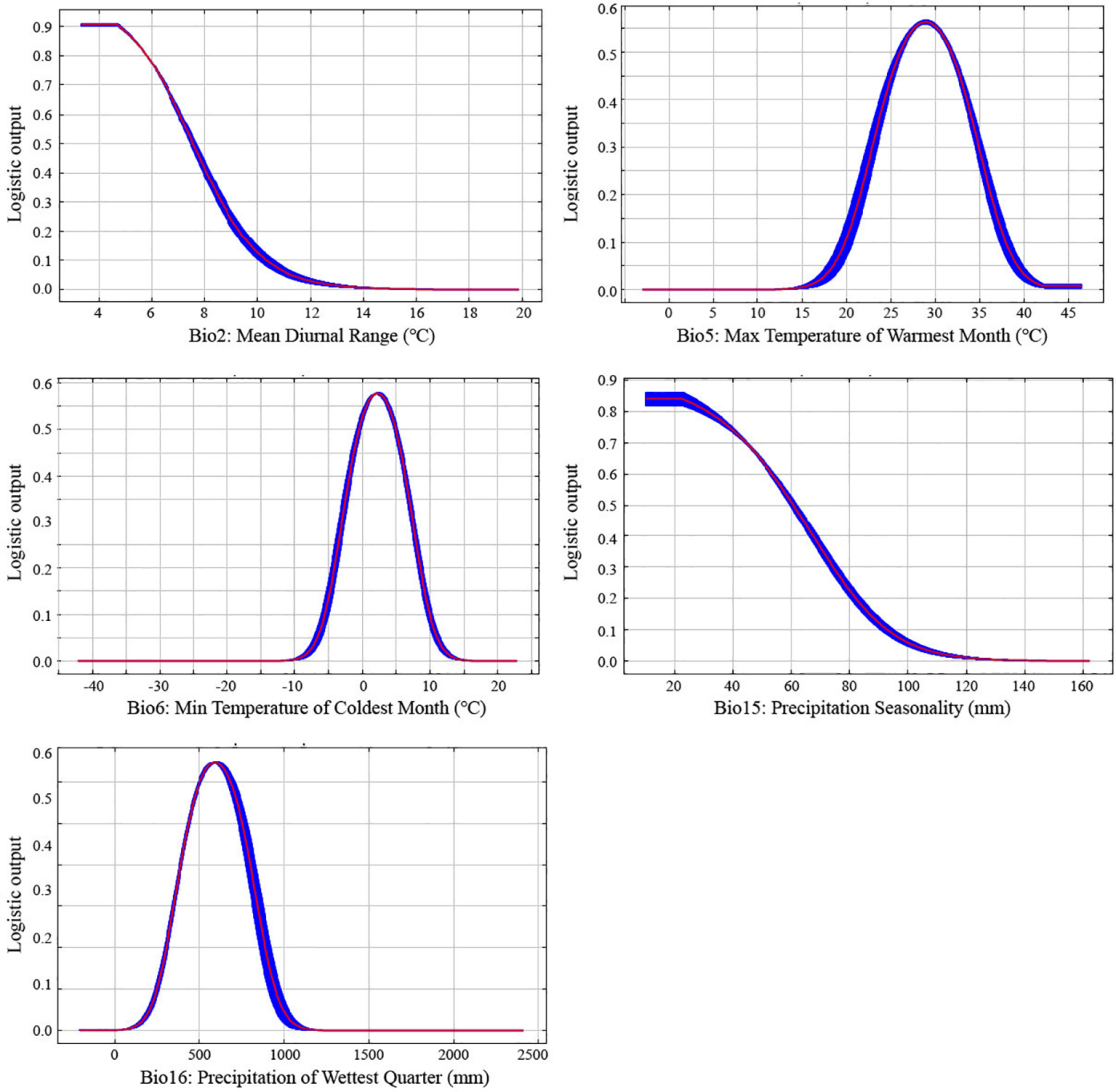
Response curves of *C. japonica* distribution relative to five environmental variables. The red line represents the mean response from 10 replicate Maxent runs, while the blue shaded area represents the mean ± one standard deviation.

**Figure 4** illustrates the relationship between five environmental variables and the probability of species incidence. The mean diurnal range (Bio2) and precipitation seasonality (Bio15) exhibited a reverse-sigmoid (negative logistic) pattern, where the probability of presence decreased sharply as variable values increased, indicating a strong limiting effect beyond certain thresholds. The maximum temperature of the warmest month (Bio5), minimum temperature of the coldest month (Bio6), and precipitation of the wettest quarter (Bio16) exhibited unimodal (parabolic) curves, reflecting optimal ranges for species occurrence. For key environmental variables, the suitable mean diurnal range (Bio2) was predicted to be < 5 C; precipitation seasonality (Bio15) was predicted to be < 20%; maximum temperature of the warmest month (Bio5) was 15 °C - 42 C; minimum temperature of the coldest month (Bio6) was -10 C -14 C; and precipitation of the wettest quarter (Bio16) was 100 mm -1100 mm. In addition, when precipitation seasonality (Bio15) exceeded 120%, the probability of *C. japonica* occurrence approached 0.

### 3.3 Potential suitable areas under current climate scenarios for *C. japonica* in China

The occurrence records compiled in this study are distributed across multiple provinces in China (**Figure 1**), reflecting the spatial coverage of available data sources rather than the strict floristic range of the species. The easternmost location of *C. japonica* is Zhoushan Archipelago in northeastern Zhejiang; the northernmost is Laoshan, Qingdao, and Shandong; the westernmost is Tengchong in Yunnan Province, and the southernmost is Shanglin in Guangxi Province. These records reflect the spatial coverage of available data sources rather than the strict floristic range of the species.

**Figure 5:**
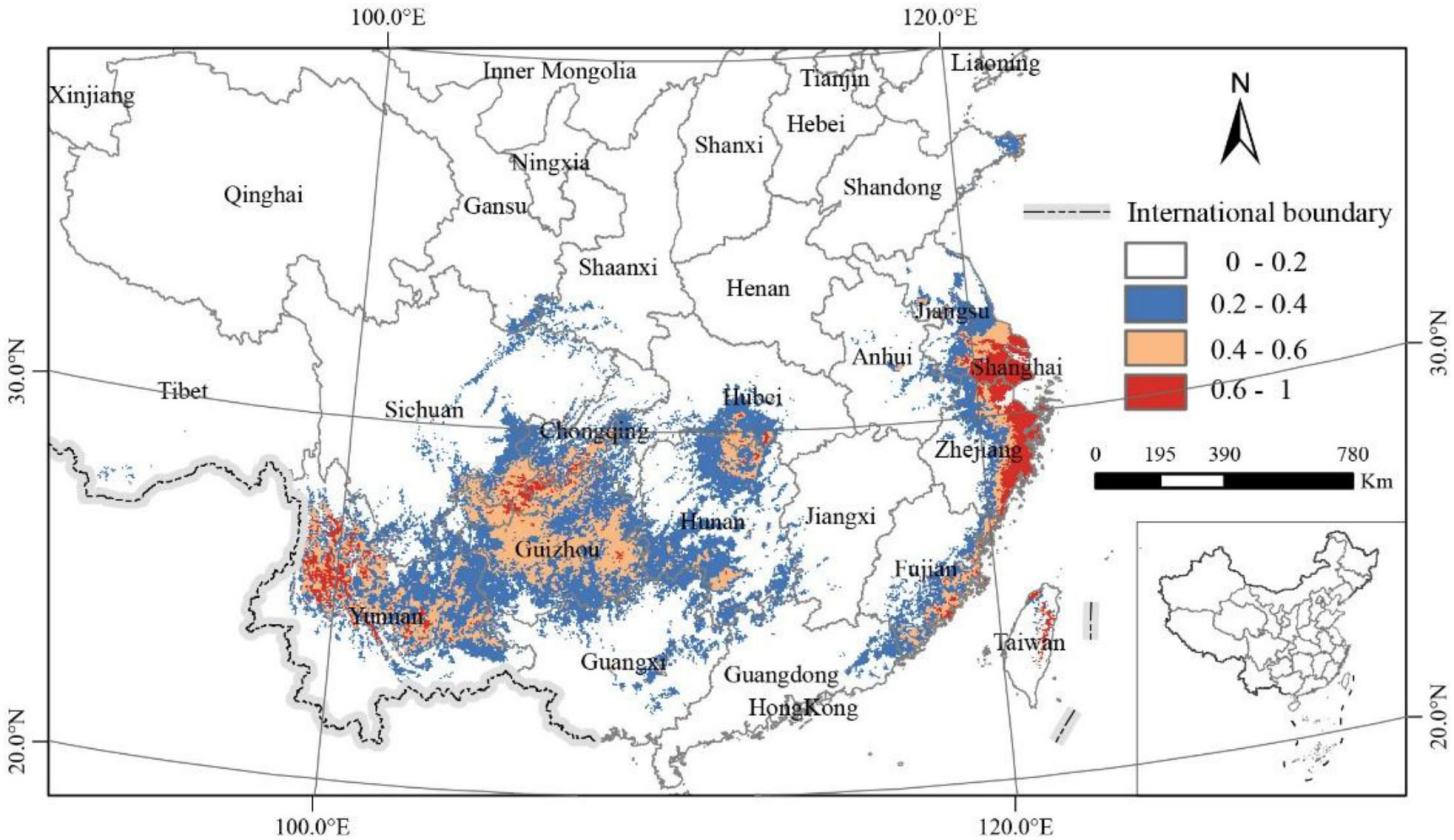
Potential biogeographical range of *C. japonica* in China under current climate conditions.

After running the Maxent model with the optimized parameters, the simulated results of potential suitable growth areas for *C. japonica* under the current climate are shown in **Figure 5**. The potential suitable areas for *C. japonica* are mainly distributed in Sichuan, Guizhou, Yunnan, Guangxi, Anhui, Fujian, Taiwan, Zhejiang, Shanghai, Jiangsu, and Shandong. The research results revealed that the predicted main suitable growth areas for *C. japonica* in China are approximately between longitudes 91.6˚E and 122.7˚E, and latitudes 22.4˚N and 37.5˚N.

The current potential distribution range of *C. japonica* is substantially larger than the current actual range. Model predictions show three newly identified high-suitability zones in Shanghai, Fujian, and Taiwan, with middle-suitability areas emerging in Hunan, Jiangsu, Chongqing, and Hubei. Additionally, low-suitability habitat was predicted along the southeastern fringe of Tibet. The total areas for no, low, middle, and high suitability are 8,652,325 km^2^, 528,348 km^2^, 228,525 km^2^, and 83,296 km^2^, respectively (**Table 2**). The total suitability areas cover 840,169 km^2^, accounting for 8.8% of China’s total land area (**Table 2**).

### 3.4 Future potential distribution of *C. japonica*

**Figure 6:**
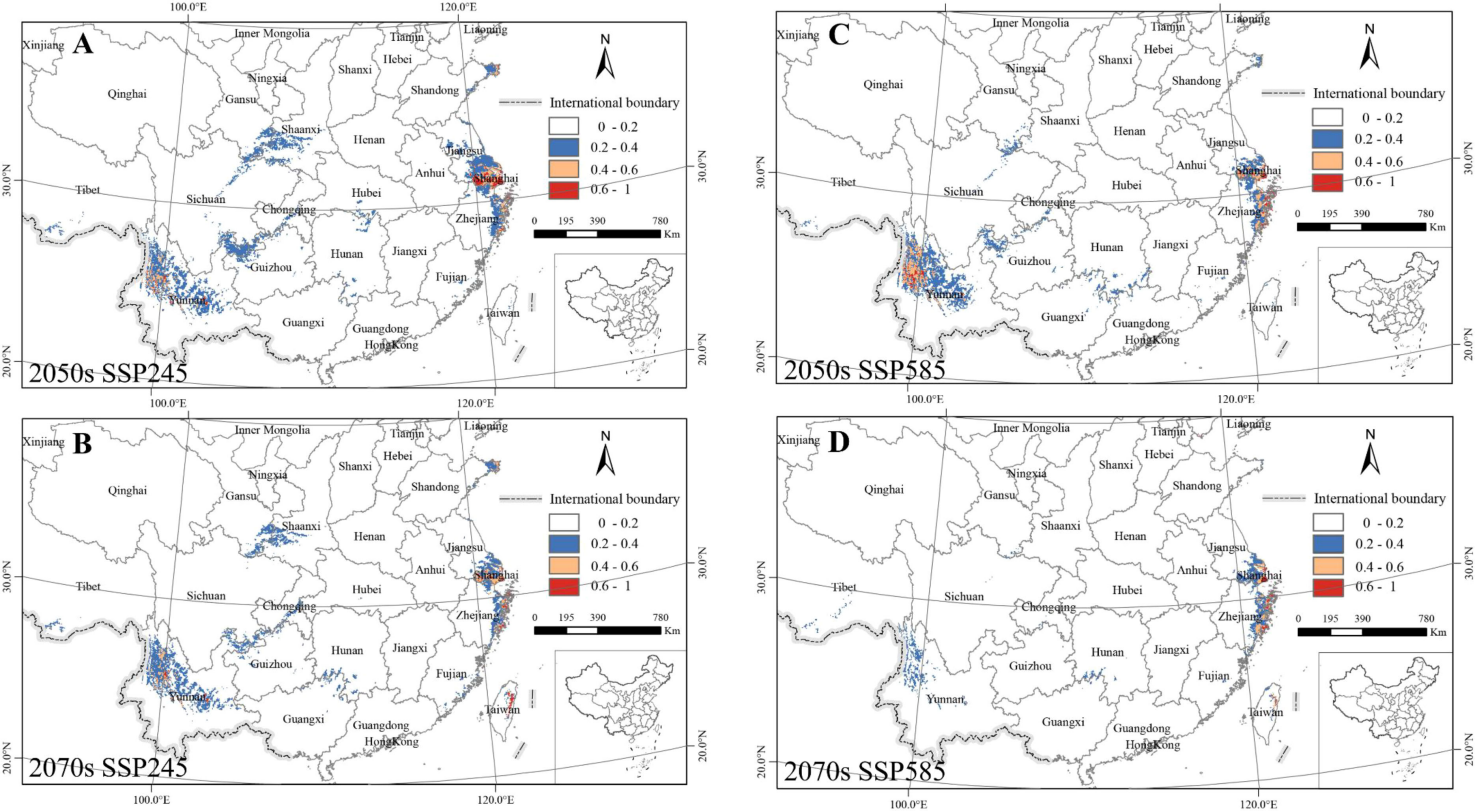
Potential distribution of *C. japonica* in China under SSP245 and SSP585 emission scenarios for the 2050s and 2070s period. MaxEnt projections were run with the default clamping option enabled to constrain predictions to the range of environmental conditions present in the training data.

Model projections under both SSP245 and SSP585 scenarios indicate a substantial contraction of suitable habitats for *C. japonica* under future climate conditions (**Figure 6**). Compared to the current suitable area (840,169 km²), the total suitable area is projected to decrease to 167,568 km² under SSP245 and 74,423 km² under SSP585 by the 2070s, representing reductions of 80.1% and 90.9%, respectively (**Table 2**). The most pronounced habitat loss is concentrated within the current core distribution regions, including Zhejiang, Shandong, and Taiwan.

Although limited expansion into northwestern regions (e.g., Shanxi and Gansu provinces) and southern Tibet is observed under the SSP245 scenario, these newly suitable areas are spatially restricted and fragmented. Under the SSP585 scenario, suitable habitats are further reduced and become highly fragmented, with only small and isolated patches remaining.

The severe contraction and fragmentation of suitable habitats constrain the interpretation of spatial redistribution patterns and preclude any reliable quantification of range shift metrics, such as centroid displacement or elevational change. Any attempt to quantify such shifts under these conditions would be unreliable and potentially misleading. Therefore, while localized changes in distribution may occur, the results do not provide sufficient evidence to confirm a consistent directional shift across future climate scenarios.

To further quantify habitat dynamics, binary suitability maps (threshold = 0.2) were compared between current and future conditions. Under SSP245 (2070s), the total suitable area decreased by 672,601 km² (80.1%). Habitat loss (areas currently suitable but becoming unsuitable) accounted for 82.3% of the current suitable area, primarily occurring in Zhejiang, Shandong, and Taiwan. In contrast, habitat gain (areas becoming newly suitable) was limited to 0.8% of the current suitable area, mainly in small patches in southern Tibet and Shanxi. The resulting loss-to-gain ratio of approximately 10:1 indicates that habitat contraction, rather than redistribution, is the dominant response of C. japonica to future climate change.

## 4 Discussion

### 4.1 Model accuracy

The reliability of species distribution models depends not only on sample size, but also on the representativeness of occurrence records in capturing the environmental space occupied by the species. In this study, the 56 occurrence records, retained after spatial thinning, cover the full geographic extent of known wild populations of *C. japonica* in China, including the northernmost (Laoshan, Shandong), southernmost (Shanglin, Guangxi), easternmost (Zhoushan, Zhejiang), and westernmost (Tengchong, Yunnan) limits (**Figure 1**). This broad spatial coverage ensures that major climatic gradients relevant to the species are adequately represented.

Importantly, these records encompass substantial variation in key climatic variables, particularly temperature and precipitation regimes, which were identified as the primary drivers of species distribution. Therefore, the dataset effectively captures the realized climatic niche of *C. japonica* within China, rather than relying solely on sample quantity. Previous studies have shown that MaxEnt can achieve reliable predictions with limited sample sizes when environmental coverage is sufficient (Pearson et al., 2007; Hernandez et al., 2006; Wisz et al., 2008).

The model exhibited high predictive performance (AUC = 0.958; **Figure 2**), further supporting the reliability of the results. However, it should be noted that AUC values may be influenced by spatial autocorrelation. In this study, the use of spatial block cross-validation (4-fold) helps to reduce this potential bias. Therefore, model performance should be interpreted cautiously, and AUC alone should not be considered definitive evidence of predictive accuracy.

In addition, the low standard deviation of test AUC across replicates (0.016), together with consistently high TSS (0.87) and CBI (0.92), indicates stable and robust model performance, suggesting that the predictive ability is not solely an artifact of spatial clustering.

Overall, the combination of adequate environmental coverage, appropriate model calibration, and multiple evaluation metrics provides a robust basis for predicting the potential distribution of *C. japonica*.

### 4.2 Current potential suitability habitats and key environmental variables

The current potential distribution of *C. japonica* is mainly concentrated in eastern and southeastern China, including Zhejiang, Fujian, Jiangxi, and parts of Shandong, which is consistent with its known natural distribution. These regions are characterized by humid subtropical climates with relatively stable temperature and precipitation regimes, providing favorable environmental conditions for the species (Fu et al., 2018; Fan et al., 2022).

Among the environmental variables, temperature-related factors, particularly the minimum temperature of the coldest month (Bio6) and mean diurnal range (Bio2), play dominant roles in shaping species distribution. The importance of Bio6 suggests that low-temperature limitation is a key factor constraining the northern boundary of *C. japonica*, while Bio2 reflects the influence of short-term temperature variability on habitat suitability. Similar patterns have been reported in other evergreen broad-leaved species, where thermal stability is critical for survival and growth (Franklin, 2010; Guisan et al., 2017).

Response curves indicate that *C. japonica* occupies a relatively narrow climatic niche, with habitat suitability declining rapidly beyond specific temperature thresholds. This pattern implies limited tolerance to environmental variability and highlights the sensitivity of the species to fluctuations in temperature conditions. Such narrow ecological tolerance has been widely recognized as an important factor influencing species distribution under current climates (Elith et al., 2011).

Precipitation-related variables also contribute to habitat suitability, although their influence is secondary compared to temperature. Adequate and stable moisture conditions are essential for *Camellia* species, which are typically adapted to warm and humid environments (Fu et al., 2018). The combined effects of temperature and precipitation therefore determine the current distribution pattern, resulting in a concentration of suitable habitats in regions with stable climatic conditions.

Overall, the distribution of *C. japonica* reflects a strong dependence on thermally stable and humid environments, indicating its sensitivity to climatic variability and providing a basis for understanding its potential response to future climate change.

### 4.3 Spatial distribution changes under future climate change

This pattern contrasts with the commonly reported poleward or elevational shifts observed in many plant species under climate warming, suggesting that species responses are strongly constrained by their ecological tolerances and climatic requirements.

Model projections show that by the 2070s, suitable habitats decrease by 80.1% under SSP245 and 90.9% under SSP585, with the most substantial losses occurring within the current core distribution regions, such as Zhejiang and Shandong (**Table 2**; **Figure 6**). Although limited expansion into northwestern regions (e.g., Shanxi and Gansu) and southern Tibet is observed under SSP245, these newly suitable areas are spatially restricted and fragmented. Under SSP585, suitable habitats are further reduced and highly fragmented, with only small and isolated patches remaining.

The severe contraction and fragmentation of suitable habitats constrain the interpretation of spatial redistribution patterns and preclude reliable quantification of range shift metrics, such as centroid displacement or elevational change. Therefore, although localized changes in distribution may occur, the results do not provide sufficient evidence to confirm a consistent directional shift across future climate scenarios.

The projected contraction of suitable habitats can be largely explained by climate-driven threshold effects identified in the model. Temperature-related variables, particularly the minimum temperature of the coldest month (Bio6) and mean diurnal range (Bio2), dominate species distribution, together accounting for more than 70% of model contribution. Response curves indicate clear threshold behavior, with habitat suitability declining sharply once temperature variability or precipitation seasonality exceeds specific limits. These interpretations are hypothesis-based and should be interpreted cautiously.

This pattern suggests a mismatch between future climatic conditions and the species’ relatively narrow ecological tolerance. Although warming may alleviate cold limitation at higher latitudes, increasing temperature variability and moisture instability within the current range are likely to exceed physiological tolerance limits, leading to net habitat loss. This interpretation is consistent with the ecological characteristics of *Camellia* species, which are adapted to stable, humid, and thermally buffered environments (Fu et al., 2018; Fan et al., 2022).

In addition, the absence of clear evidence for range shift may be related to dispersal limitation and potential constraints on colonization of newly suitable areas. Biotic interactions, such as plant–soil feedbacks, may further restrict establishment in these regions, although these processes were not explicitly modeled and should be interpreted cautiously (Van der Putten et al., 2013). Future studies integrating species distribution models with dispersal dynamics and biotic interactions are needed to better evaluate the realized responses of *C. japonica* to climate change.

### 4.4 Limitations and future research of this study

Species distributions are influenced by multiple interacting factors, including climate, soil conditions, human activities, geographic barriers, and biotic interactions (Gallardo and Aldridge, 2013; Xie et al., 2021). At broad spatial scales, climate is generally considered the dominant driver of species distributions. Accordingly, this study focused on climatic variables to model the potential distribution of *C. japonica*. However, the exclusion of non-climatic factors, such as soil properties and anthropogenic influences, represents an important limitation. The lack of consistent, high-resolution datasets for these variables under future climate scenarios further constrains their incorporation into predictive models (Schillaci et al., 2017; Zhang et al., 2018, 2024). As a result, the predicted suitable areas may be overestimated in regions where non-climatic constraints are strong.

Second, uncertainty may arise from the spatial resolution mismatch between occurrence records and environmental variables. In this study, some occurrence data were derived from county-level records, and county centroids were used when precise geographic coordinates were unavailable. Although spatial thinning was applied to reduce sampling bias, this approach may introduce positional uncertainty when aligning occurrence points with climate grid cells (~5 km resolution). While such methods are commonly used in species distribution modeling (Xie et al., 2021; Gebrewahid et al., 2020), future studies should prioritize the use of high-precision GPS data to improve spatial accuracy.

Third, the occurrence data used in this study were restricted to China, whereas *C. japonica* has a broader natural distribution across East Asia, including Japan and Korea. This geographic limitation may lead to partial representation of the species’ full climatic niche. However, this study specifically focuses on remnant wild populations within China, which are geographically restricted and of particular conservation concern. The selected occurrence records capture a wide range of climatic conditions within the study area, thereby reducing, though not entirely eliminating, the potential bias associated with niche truncation. Future studies incorporating occurrence data from the entire East Asian range would help to improve the robustness and generality of model predictions.

Fourth, uncertainties also arise in future climate projections. Although we used the ensemble mean of five CMIP6 climate models and applied the clamping function in MaxEnt to limit unrealistic extrapolation, projections may still involve novel climatic conditions beyond the range of the training data. Such extrapolation introduces unavoidable uncertainty in predicting future distributions. Additional approaches, such as Multivariate Environmental Similarity Surface (MESS) analysis, could be applied in future studies to better identify areas of high extrapolation risk and to further evaluate prediction reliability.

Future research should aim to integrate multiple environmental drivers, including soil, land use, and biotic interactions, and to incorporate dynamic or process-based modeling approaches that explicitly consider dispersal processes and ecological interactions. These improvements would enhance the ecological realism and predictive reliability of species distribution models under future climate change scenarios.

## 5 Conclusion

This study used the MaxEnt model to evaluate the potential distribution of *C. japonica* in China under current and future climate scenarios. Temperature-related variables, particularly the minimum temperature of the coldest month (Bio6) and mean diurnal range (Bio2), were identified as the primary factors shaping its distribution.

Model projections indicate a substantial contraction of suitable habitats under future climate scenarios, with reductions exceeding 80% by the 2070s. This contraction is concentrated within the current core range, while newly suitable areas are limited and fragmented. Due to this fragmentation, range shift could not be reliably quantified, and the results do not provide sufficient evidence to confirm a consistent directional shift.

Overall, habitat contraction rather than redistribution appears to be the dominant response of *C. japonica* to climate change. These findings highlight the vulnerability of remnant wild populations and emphasize the importance of targeted conservation strategies focusing on climatically stable refugia.

## Data Availability

Publicly available datasets were analyzed in this study. This data can be found here: the Chinese Virtual Herbarium (CVH, http://www.cvh.ac.cn/), National Specimen Information Infrastructure (NSII, http://www.nsii.org.cn/), the Global Biodiversity Information Facility (GBIF, http://www.gbif.org), Web of Science, and China National Knowledge Infrastructure (CNKI, http://oversea.cnki.net/tra). When occurrence records lacked precise geographic coordinates, Google Earth (http://ditu.google.cn/) was used to find latitude and longitude.
