# Supplementary material for: Projected habitat contraction of Camellia japonica under climate change in China based on MaxEnt modeling
Source: Front Plant Sci. 2026 May 18;17:1800676. doi: 10.3389/fpls.2026.1800676 (PMC13223104; doi:10.3389/fpls.2026.1800676)
Supplement: Supplementary file 1 [file Table1.docx]

**Supplementary Table S1**. Variance Inflation Factor (VIF) values of selected environmental variables with low multicollinearity (VIF < 10).

| **Variable** | **Description** | **VIF** |
| --- | --- | --- |
| Bio1 | Annual Mean Temperature | 3.11 |
| Bio6 | Min Temperature of Coldest Month | 1.63 |
| Bio13 | Precipitation of Wettest Month | 5.69 |
| Bio14 | Precipitation of Driest Month | 3.77 |

Note: VIF values are presented for a subset of variables that met the VIF < 10 criterion to demonstrate low multicollinearity conditions. VIF was used as a diagnostic indicator rather than a strict exclusion threshold for all variables.
